# Regulation of glutamatergic signalling by PACAP in the mammalian suprachiasmatic nucleus

**DOI:** 10.1186/1471-2202-7-15

**Published:** 2006-02-16

**Authors:** Stephan Michel, Jason Itri, Jung H Han, Kathryn Gniotczynski, Christopher S Colwell

**Affiliations:** 1 Department of Molecular Cell Biology, Laboratory for Neurophysiology, Leiden University Medical Center, P.O. Box 9600, 2300 RC Leiden, The Netherlands; 2 Department of Psychiatry and Biobehavioral Sciences, University of California – Los Angeles, 760 Westwood Plaza, Los Angeles, California 90024-1759, USA

## Abstract

**Background:**

Previous studies indicate that light information reaches the suprachiasmatic nucleus (SCN) through a subpopulation of retinal ganglion cells that contain both glutamate and pituitary adenylyl cyclase activating peptide (PACAP). While the role of glutamate in this pathway has been well studied, the involvement of PACAP and its receptors are only beginning to be understood. Speculating that PACAP may function to modulate how neurons in the suprachiasmatic nucleus respond to glutamate, we used electrophysiological and calcium imaging tools to examine possible cellular interactions between these co-transmitters.

**Results:**

Exogenous application of PACAP increased both the amplitude and frequency of spontaneous excitatory postsynaptic currents recorded from SCN neurons in a mouse brain slice preparation. PACAP also increased the magnitude of AMPA-evoked currents through a mechanism mediated by PAC1 receptors and the adenylyl cyclase-signalling cascade. This enhancement of excitatory currents was not limited to those evoked by AMPA as the magnitude of NMDA currents were also enhanced by application of PACAP. Furthermore, PACAP enhanced AMPA and NMDA evoked calcium transients while PACAP alone produced very little change in resting calcium in most mouse SCN neurons. Finally, in rat SCN neurons, exogenous PACAP enhanced AMPA evoked currents and calcium transients as well evoked robust calcium transients on its own.

**Conclusion:**

The results reported here show that PACAP is a potent modulator of glutamatergic signalling within the SCN in the early night.

## Background

In mammals, the neural structure responsible for most circadian behaviours can be localized to a bilaterally paired structure in the hypothalamus, the suprachiasmatic nucleus (SCN). These SCN neurons must be synchronized to each other as well as to the environment in order to function adaptively. The daily cycle of light and dark is the dominant environmental cue responsible for synchronizing this biological timing system to the environment. The SCN receives photic information directly through a monosynaptic projection from the retina known as the retinal hypothalamic tract (RHT). The RHT comprises a distinct subset of retinal ganglion cells that contain a novel photopigment melanopsin and are directly light-sensitive [1,2]. There is a variety of evidence that the amino acid glutamate is a transmitter at the RHT/SCN synaptic connection and that this transmitter plays a critical role in mediating photic regulation of the circadian system [3,4]. One of the main post -synaptic consequences of glutamate receptor activation within the SCN is an increase in intracellular calcium (Ca^2+^) [5,6]. The signal transduction events after Ca^2+ ^release are beginning to be understood and include the release of nitric oxide, activation of the Ras/MAP kinase cascade, and ultimately changes in gene expression [7-9].

The neuropeptide PACAP has emerged as a likely retinal messenger to the SCN, acting in concert with glutamate to communicate with the SCN. PACAP-like immunoreactivity is found in terminals of retinal ganglion neurons innervating the SCN [10,11] and two of the receptors sensitive to PACAP (PAC1 and VPAC2) are expressed in the SCN [12-15]. In the SCN, application of PACAP results in Ca^2+ ^transients [16,17], activation of the MAPK signalling cascade [17,18], and changes in gene expression [18-21]. At a systems level, applica tion of PACAP can shift the phase [22,23] or alter the magnitude of glutamate -induced phase shifts [24] of the circadian rhythm of SCN neuronal firing in a brain slice preparation. Similarly, microinjections of PACAP into the SCN region *in vivo *can cause phase shifts [23,25-27]. Administration of a PACAP receptor antagonist or an antibody against PACAP attenuates light-induced phase delays [27]. The circadian system of mice deficient in the PAC1 receptor [28], the VPAC2 receptor [29], or PACAP [30], each exhibited altered behavioural responses to light. In our own work, we have found that PACAP-deficient mice exhibit a selective loss in the magnitude of light-induced phase advances and delays [31]. Given this previous data, we became interested in understanding more about PACAP/glutamate interactions at the cellular level in the night.

## Results

### PACAP enhanced excitatory synaptic transmission measured in mouse SCN neurons

**Figure 1:**
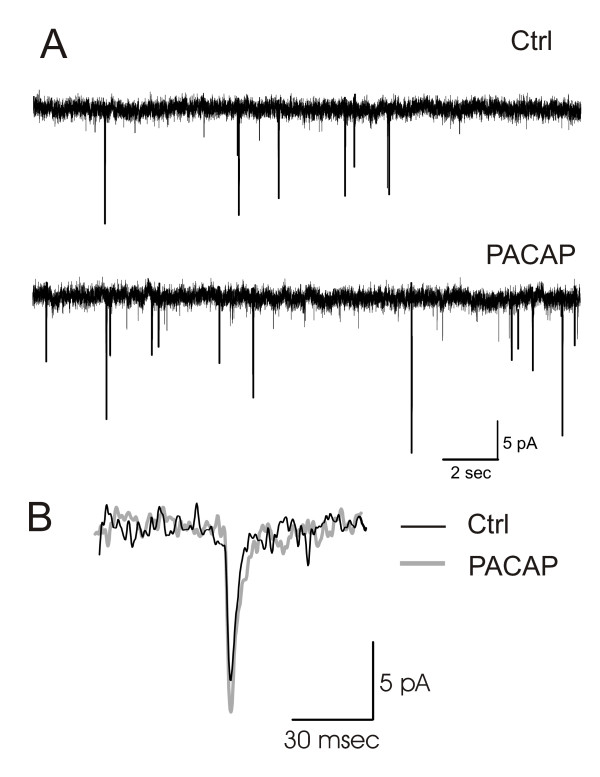
**PACAP enhances the frequency and amplitude of sEPSCs recorded from mouse SCN neurons**. Whole cell patch clamp recording techniques were used to measure the sEPSCs in ventral SCN neurons during the night (ZT 15–17). All experiments were carried out in presence of TTX (0.5 μM) and bicuculline (25 μM). **(A) **Top panel provides an example of sEPSCs recorded before and after treatment with PACAP (10 nM, 240 sec). PACAP increased the frequency (4 out of 6 neurons) and the amplitude (6 out of 6 neurons) of the sEPSCs. **(B) **Average sEPSC waveform recorded in an SCN neuron before and after treatment with PACAP (10 nM, 240 sec). PACAP increased the sEPSC amplitude by 20% ± 5 %.

PACAP is co-expressed with glutamate in at least some of the retinal ganglion cell population that innervates the SCN [10,11]. Whole -cell voltage -clamp recording techniques were used to test the hypothesis that PACAP may function to modulate spontaneous excitatory postsynaptic currents (sEPSCs) in ventral SCN neurons during the night (ZT 15–17; Fig. 1). The mean frequency and the mean amplitude of sEPSCs recorded at a holding potential of -70 mV were 0.17 ± 0.03 Hz and -10.9 ± 0.8 pA, respectively (*n *= 6). These sEPSCs were recorded in the presence of TTX and bicuculline. The sEPSCs were completely abolished with CNQX (25 μM, 5 of 5 neurons tested, data not shown), indicating that they are largely mediated by AMPA/KA GluRs. Application of PACAP (10 nM) increased the sEPSC amplitude by 20% ± 5% (*n *= 6, *P *< 0.01) in all ventral SCN neurons examined. PACAP also increased the sEPSC frequency in 4 of 6 cells (Control: 0.17 ± 0.03 Hz; PACAP: 0.42 ± 0.2 Hz, *P *< 0.05). Thus PACAP can increase both sEPSC frequency and amplitude, suggesting that PACAP can modulate both pre-synaptic release as well as post-synaptic sensitivity to glutamate.

### PACAP enhanced AMPA-evoked currents recorded in mouse SCN neurons

**Figure 2:**
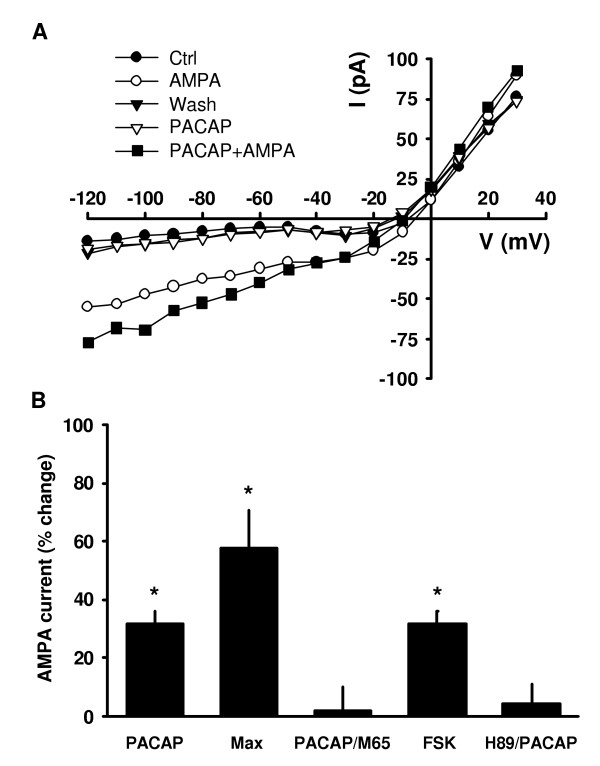
**PACAP enhanced AMPA currents in mouse SCN neurons**. Whole cell patch clamp recording techniques were used to directly measure currents evoked by AMPA in ventral SCN neurons during the night (ZT 15–17). The voltage -dependence of the AMPA-evoked currents was measured by moving the membrane potential of the cell through a series of voltage -steps before, during, and after treatment with AMPA (25 μM) in the bath. **(A) **By itself, PACAP (10 nM, 240 sec) did not activate voltage-dependent currents, however, PACAP did increase the magnitude of AMPA-evoked currents. Top panel shows current-voltage relationships for peak current during AMPA treatment recorded using this protocol before and after treatment with PACAP (10 nM, 240 sec). **(B) **Histograms demonstrating that the effects of PACAP were mimicked by the PAC1 receptor agonist maxadilan and blocked by the antagonist M65. By itself, M65 did not alter the magnitude of the AMPA current (data not shown). In addition, effects of PACAP were mimicked by the AC activator FSK and blocked by the PKA inhibitor H89. By itself, H89 did not alter the magnitude of the AMPA current (data not shown). Asterisks indicate values that are significantly different than those of the AMPA-treated group (*P *< 0.05).

To directly test the hypothesis that PACAP modulates the post-synaptic response of SCN neurons to glutamatergic stimulation, whole cell patch clamp recording techniques were used to directly measure currents evoked by AMPA (25 μM) in ventral SCN neurons (Fig. 2). Most SCN neurons (91%; 78 out of 86 neurons tested) exhibited AMPA-evoked currents. These inward currents exhibited a linear voltage-dependency with reversal potentials around 0 mV. AMPA-evoked currents were stable (<5% change in amplitude) over the course of 30 min of recording (*n *= 6; data not shown) and were eliminated by the addition of the AMPA/KA GluR antagonist CNQX (25 μM; *n *= 6; data not shown). These experiments were performed in the presence of TEA (10 mM), TTX (0.5 μM) and Cd^2+ ^(25 μM) to block most voltage dependent ionic currents. The bath application of AMPA (25 μM) produced a normalized peak current of -4.6 ± 0.6 pA/pF (*n *= 27) in ventral SCN neurons recorded in the night (ZT 15–17). Pre-treatment with PACAP (10 nM, 240 sec) significantly enhanced the magnitude of AMPA-evoked currents in SCN neurons examined (8 out of 10 neurons, 34% ± 5% increase in peak current, *P *< 0.01, Fig. 2). Pre-treatment with a higher concentration of PACAP (100 nM, 240 sec) did not result in a greater enhancement of the magnitude of AMPA -evoked currents in SCN neurons examined (5 out of 7 neurons, 30% ± 5% increase in peak current). Lower concentrations of PACAP did not produce significant changes in the magnitude of the AMPA response (0.1 nM PACAP: 5% ± 2%, *n *= 5; 1.0 nM PACAP: 13 ± 10%, *n *= 6). By itself, PACAP did not evoke any measurable current in SCN neurons held at a range of different voltages (-120 mV to 30 mV; *n *= 14).

In the next set of experiments, we wanted to determine if this e ffect of PACAP is mediated by PAC1 receptors and a regulation of the adenylyl cyclase (AC) signalling cascade (Fig. 2B). The PAC1 receptor agonist maxadilan (10 nM, 240 sec) enhanced the peak current evoked by AMPA (58% ± 13%, *n *= 8; *P *< 0.01) whereas pretreatment with the PAC1 antagonist M65 prevented the stimulatory effect of PACAP (10 nM) on AMPA currents (2% ± 8%, *n *= 7). PAC1 receptors are positively coupled to AC activity and additional experiments were designed to investigate if PACAP enhancement of AMPA currents could be mediated through this mechanism. The application of an AC activator, forskolin (FSK, 10 μM, 240 sec) increased the magnitude of the AMPA currents (8 out of 11 neurons; 24% ± 3%; *P *< 0.05). By itself, application of the PKA inhibitor, H89 (20 μM, 240 sec), alone did not significantly alter the magnitude of the AMPA currents (1% ± 7%, *n *= 6, *P *> 0.05); however, in the presence of H89, PACAP no longer produced a significant change in the magnitude of the AMPA current (4% ± 6%, *n *= 8, *P *> 0.05). This data was all collected in ventral SCN neurons recorded in the night (ZT 15–17). This data suggests that PACAP can modulate AMPA currents through a PAC1 receptor mediated activation of the AC/PKA signalling pathway.

### PACAP enhanced NMDA-evoked currents recorded in mouse SCN neurons

**Figure 3:**
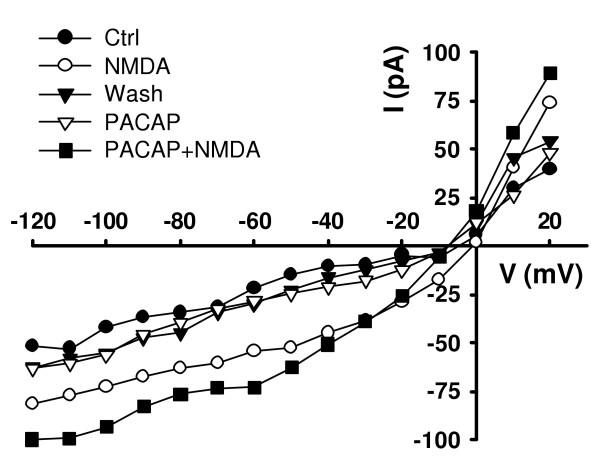
**PACAP enhanced NMDA currents in mouse SCN neurons**. Whole cell patch clamp recording techniques were used to directly measure currents evoked by NMDA in ventral SCN neurons during the night (ZT 15–17). The voltage -dependence of the NMDA-evoked currents was measured by moving the membrane potential of the cell through a series of voltage -steps before, during, and after treatment with NMDA (25 μM) in the bath. By itself, PACAP (10 nM, 240 sec) did not activate voltage-dependent currents, however, PACAP did increase the magnitude of NMDA-evoked currents. Current-voltage relationships for peak current during NMDA treatment recorded using this protocol before and after treatment with PACAP (10 nM).

PACAP can potentiate NMDA currents in acutely disassociated hamster SCN neurons [23] and we sought to determine if PACAP has the same effect in ventral SCN neurons in mice (Fig. 3). Whole cell patch clamp recording techniques were used to directly measure currents evoked by NMDA (25 μM) in ventral SCN neurons recorded during the night (ZT 15–17). These experiments were performed in the presence of TEA (10 mM), TTX (0.5 μM) and Cd^2+ ^(25 μM) in the bath. Most SCN neurons (75%) exhibited NMDA-evoked currents (*n *= 69). These inward currents were voltage-dependent, i.e. NMDA-evoked currents peaked when the cell was held at a membrane potential between -20 and -40 mV. NMDA-evoked currents were stable (<5% change in amplitude) over the course of 30 min of recording (*n *= 6; data not shown) and were reduced 85% by the addition of the NMDA antagonist AP5 (50 μM; *n *= 8, data not shown). The bath application of NMDA (25 μM) produced a normalized peak current of -2.3 ± 0.2 pA/pF (*n *= 8) in ventral SCN neurons recorded in the night. Treatment with PACAP (10 nM, 240 sec) also significantly enhanced the magnitude of NMDA-evoked currents in SCN neurons examined (6 out of 8 neurons, 106% ± 31% increase in peak current, *P *< 0.01, Fig. 3). The application of FSK (10 μM, 240 sec) also increased the magnitude of the NMDA currents (8 out of 11 neurons; 24% ± 3%; P < 0.05). In the presence of H89, PACAP no longer produced a significant change in the magnitude of the NMDA current (-10% ± 3%, n = 5, *P *> 0.05).

### PACAP enhanced AMPA and NMDA evoked Ca^2+ ^transients in mouse SCN neurons

**Figure 4:**
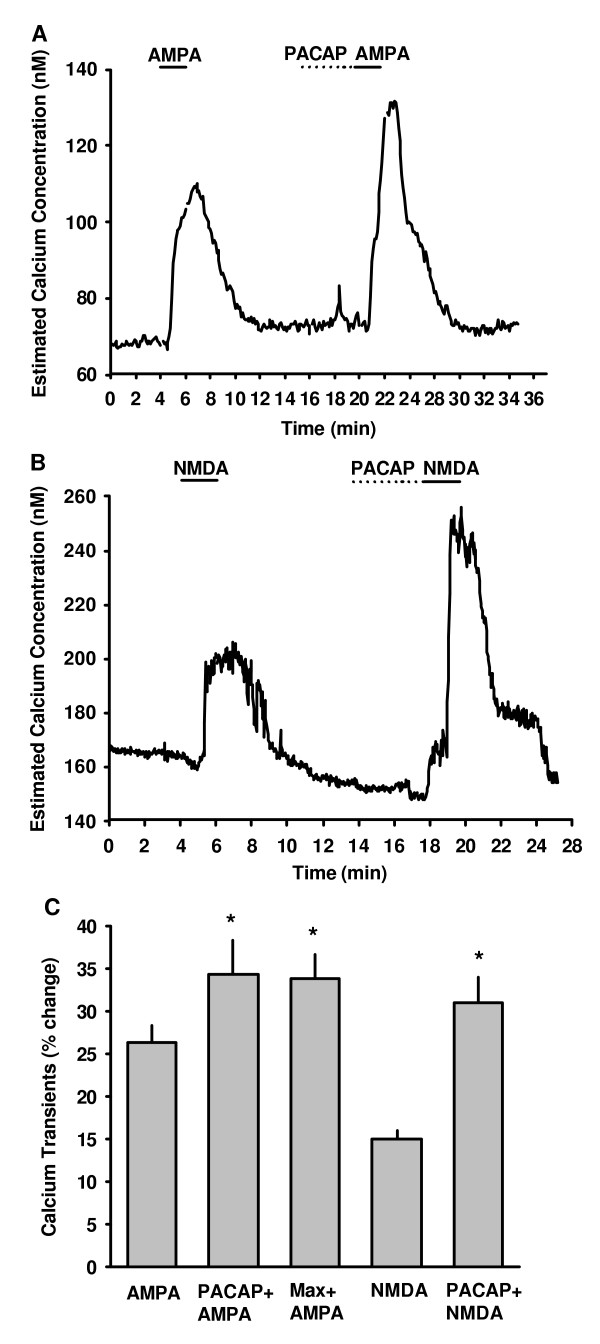
**PACAP enhanced AMPA- and NMDA-evoked Ca^2+ ^responses in a subset of mouse SCN neurons**. Optical imaging techniques were used to estimate Ca^2+ ^responses to AMPA and NMDA in ventral SCN neurons during the night (ZT 15–17). **(A) **AMPA (25 μM)-induced Ca^2+ ^transients were also enhanced by the pre-treatment with PACAP (10 nM, 240 sec). **(B) **NMDA (25 μM)-induced Ca^2+ ^transients were also enhanced by the pre-treatment with PACAP (10 nM, 240 sec). **(C) **Histograms summarizing PACAP modulation of AMPA and NMDA-evoked Ca^2+ ^transients in the SCN. The enhancement of AMPA-evoked Ca^2+ ^transients was mimicked by the PAC1 receptor agonist maxadilan (Max, 100 nM, 240 sec). This data was collected in the presence of TTX. Asterisks indicate values that are significantly greater than those of the AMPA- or NMDA-treated groups (*P *< 0.05)

One of the main postsynaptic consequences of glutamatergic stimulation of neurons in the SCN is the induction of a Ca^2+ ^transient [6,32,33]. Previous studies have demonstrated that PACAP alone can increase Ca^2+ ^as well as modulate glutamatergic evoked responses in cultured rat SCN neurons [16,17,34]. In order to test the hypothesis that PACAP regulates AMPA – and NMDA-induced Ca^2+ ^transients in ventral SCN neurons during the night (ZT 15–17), a bulk loading procedure was used to load cells with a membrane permeable form of the Ca^2+ ^indicator dye fura2. Cells that exhibited uneven loading due to dye sequestration were not included in the data set. Small cell types including glia were easily identified and were also excluded in the data set. These experiments were carried out in the presence of TTX. By itself, PACAP (100 nM, 240 sec) produced an average increase in Ca^2+ ^of 6% ± 1% (*n *= 177) whereas a lower concentration of PACAP (10 nM, 240 sec) produced an average increase in Ca^2+ ^of 2% ± 1% (*n *= 60). Lower concentrations of PACAP (1 and 0.1 nM, 240 sec) did not alter Ca^2+ ^levels within SCN neurons (data not shown). While most cells (approximately 70%) in the ventral SCN region did not respond to PACAP, there was a subpopulation of cells that exhibited a robust Ca^2+ ^response to the bath application of PACAP. In this subpopulation within the ventral SCN (49 out of 177 cells sampled), PACAP (100 nM, 240 sec) caused mean peak changes in Ca^2+ ^of 19% ± 1%. In addition, PACAP pre-treatment significantly enhanced SCN cells' response to AMPA (Fig. 4A). Within the ventral SCN (*n *= 99), bath application of AMPA (25 μM, 120 sec) alone increased Ca^2+ ^levels by 26% ± 2% whereas the same treatment increased Ca^2+ ^levels by 34% ± 4% after pre-treatment with PACAP (10 nM, 240 sec; *P *< 0.05; Fig. 4B). The effects of PACAP on AMPA were mimicked by the PAC1 agonist maxadilan (100 nM, 240 sec) (Fig. 4C; maxadilan + AMPA: 34% ± 3% increase; maxadilan: 1.6% ± 0.3% increase; *n *= 86). Finally, PACAP pre-treatment significantly enhanced SCN cells' response to NMDA (Fig. 4B). Within the ventral SCN (*n *= 39), bath application of NMD A (25 μM, 120 sec) alone increased Ca^2+ ^levels by 15% ± 1% whereas the same treatment increased Ca^2+ ^levels by 31% ± 3% after treatment with PACAP (10 nM, 240 sec; *P *< 0.001).

### PACAP enhanced AMPA currents and AMPA-evoked Ca^2+ ^transients in rat SCN neurons

**Figure 5:**
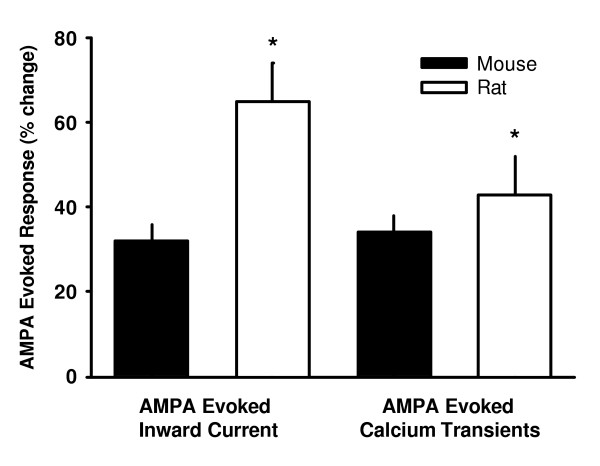
**Comparison between AMPA-evoked responses in SCN neurons of rats and mice**. Optical imaging techniques were used to estimate Ca^2+ ^responses to A MPA in ventral SCN neurons during the night (ZT 15–17). Application of PACAP increased AMPA-evoked currents and Ca^2+ ^responses in ventral SCN region of mice and rats. The left two histograms compare PACAP (10 nM, 240 sec) enhancement of AMPA (25 μM, 120 sec) induced currents in mouse and rat. The right two histograms compare PACAP (10 nM, 240 sec) enhancement of AMPA (25 μM, 120 sec) induced Ca^2+ ^transients in mouse and rat. Pretreatment with PACAP significantly increased AMPA evoked currents and Ca^2+ ^transients in both mice and rats. In both cases, the effects of PACAP were significantly larger in the rat. Asterisks indicate values that are significantly greater in the rat then in the mouse (*P *< 0.05). Data is shown as means ± SE.

Previous studies in cultured rat SCN neurons have demonstrated that PACAP alone can increase Ca^2+ ^as well as modulate glutamatergic evoked responses [16,17]. In the final set of experiments, we sought to determine if the PACAP regulation of AMPA currents and Ca^2+ ^transients that we described in mice were also present in the rat preparation (Fig. 5). The experimental conditions for experiments with the rat were the same as described for the mouse above with the data collected from ventral SCN neurons during the night (ZT 15–17). The bath application of AMPA (25 μM) produced a normalized peak current of -7.2 ± 0. 8 pA/pF (*n *= 15). Treatment with PACAP (10 nM) significantly enhanced the magnitude of AMPA -evoked currents in these cells (-8.5 ± 0. 9 pA/pF, *n *= 15, *P *< 0.01). Application of PACAP alone (100 nM, 240 sec) produced measurable Ca^2+ ^transients in most SCN neurons (33 out of 42 neurons) with an average increase in Ca^2+ ^of 85% ± 35%. Finally, pre-treatment with PACAP significantly enhanced AMPA-evoked Ca^2+ ^transients within the rat SCN. Bath application of AMPA (25 μM, 120 sec) alone increased Ca^2+ ^levels by 28% ± 6% whereas the same treatment increased Ca^2+ ^levels by 43% ± 8% after treatment with PACAP (10 nM, 240 sec; *n *= 22; *P *< 0.05). Therefore, while PACAP enhanced AMPA evoked currents and Ca^2+ ^transients in both species, the magnitude of PACAP's actions were greater in the rat.

## Discussion

The neuropeptide PACAP has emerged as a likely retinal messenger to the SCN acting in concert with glutamate to communicate with the SCN. Understanding the cellular mecha nisms by which PACAP regulates SCN neurons is a critical step toward the development of a mechanistic explanation for the role of this peptide in the mammalian circadian system. The data reported here demonstrate that PACAP is a potent modulator of glutamatergic signalling within the SCN during the night. First, we found that PACAP increases the sEPSC frequency recorded in SCN in the presence of TTX. Each miniature synaptic current is thought to result from the spontaneous fusion of an individual synaptic vesicle with the pre-synaptic membrane subsequently resulting in quantal release of transmitter molecules. Changes in the frequency at which this process occurs are normally associated with alterations in the pre -synaptic release process while changes in the amplitude of the currents reflect postsynaptic changes in receptor sensitivity or ionic driving force. The finding that PACAP changed the sEPSC frequency indicates a pre-synaptic mechanism. Interestingly, the PAC1 receptor mRNA and protein are expressed in retinal ganglion cells [35] and may thus be in the neurons that form the RHT. At least one previous study found evidence to suggest that PACAP can act presynaptically to increase the frequency of excitatory postsynaptic potentials in the hippocampus [36]. In addition to the enhanced presynaptic release of glutamate, the observation that PACAP can directly increase the amplitude of sEPSCs is consistent with post-synaptic regulation.

We then directly demonstrated that application of PACAP enhances the magnitude of the currents evoked by application of AMPA and NMDA. This later data complements a previous study by Harrington and colleagues [23] demonstrating that PACAP can enhance NMDA currents in acutely disassociated hamster SCN neurons. We assume that glutamate receptor mediated Ca^2+ ^transients are likely to be critically involved in the regulation of transcriptional events needed to generate phase shifts of circadian rhythms. Therefore, we examined the effect of PACAP on Ca^2+ ^levels in SCN neurons. PACAP elicits marked Ca^2+ ^transients in about 30% of the ventral SCN cells that we examined. This effect was quite modest in the mouse and not statistically significant but was much more robust in the rat. PACAP-mediated increases in cytosolic Ca^2+ ^have been observed previously in cultured rat SCN neurons and have been found due to the release of Ca^2+ ^from intracellular stores [16,34]. PACAP also enhanced the magnitude of the Ca^2+ ^transient evoked by stimulation with AMPA and NMDA. PACAP potentiation of AMPA-evoked Ca^2+ ^transients has previously been demonstrated from cultured rat SCN neurons [17,34].

PACAP is a member of the glucagon/vasoactive intestinal peptide/secretin/growth hormone -releasing family of structurally related peptides [38]. The neuromodulatory actions of PACAP are mediated by three receptor subtypes: PAC1, VPAC1, and VPAC2 [39]. The PAC1 receptor is more selective for PACAP while the VPAC1 and VPAC2 receptors can be potently activated by VIP or PACAP [38]. Both PAC1 and VPAC2 mRNAs are expressed within the rat SCN [12-15]. In the present study, we found that the PAC1 receptor agonist maxadilan [40] mimicked the effects of PACAP on AMPA-evoked currents and Ca^2+ ^transients whereas pretreatment with the PAC1 antagonist M65 [41,42] prevented the stimulatory effect of PACAP. As is the case with many receptor agonists, maxadilan produced a stronger effect then equal molar concentrations of PACAP itself [43].

Application of maxadilan alone has been previously shown to evoke changes in the firing rate of 40% of SCN neurons examined and activations to this agonist were not altered by a VPAC2 antagonist (PG 99–465) [44]. A recent study found that PAC1 mRNA was present in nearly half (45%) of the VIP expressing neurons in the rat SCN [15]. This frequency of receptor expression fits reasonably well with our Ca^2+ ^imaging measurements in the ventral SCN in which about half the SCN cells sampled exhibited a PACAP enhancement of AMPA evoked responses. Similarly, previous physiological studies are also consistent with the PAC1 receptor mediating many of the effects of PACAP in the SCN [16,22,45]. Thus, the available evidence indicates that the PAC1 receptor is responsible for mediating the effects of PACAP on SCN neurons.

The PAC1 receptor has several possible splice variants that allow the receptor to couple to different second messenger signalling cascades [46]. A PCR analys is of the mRNA isolated from the rat SCN found evidence for the expression of two PAC1 isoforms including the PAC1_null _splice variant [47]. This form of the receptor can potently activate AC through Gs in transfected cells [38,46]. NMDA and AMPA receptors are known to be subject to a wide range of regulatory/modulatory influences including serine/threonine kinases [48,49]. Therefore, we examined the hypothesis that PACAP regulates AMPA receptors through an activation of the AC/PKA signalling cascade. We found that the application of an AC activator (FSK) mimicked the enhancement of AMPA currents produced by PACAP. Pretreatment with a PKA inhibitor (H89) prevented the enhancement of AMPA currents by PACAP but, by itself, H89 did not alter these currents. The simplest explanation for our data is that PACAP modulate glutamatergic signalling through a PAC1 receptor mediated activation of AC activity. However, less direct effects are also possible. For example, PACAP increases expression of scaffolding proteins Homer1 and Rack1 and both of these proteins have been shown to play a role in the regulation of glutamateric synaptic transmission [20,50]. Furthermore, previous work by Dziema and Obrietan [17] suggests that PACAP enhances AMPA-evoked Ca^2+ ^transients through a regulation of L -type Ca^2+ ^currents mediated by MAPK signal transduction cascade. In contrast, we still saw PACAP-enhancement of AMPA currents even when voltage-gated Ca^2+ ^currents were blocked by cadmium. Thus PACAP appears to utilize a variety of mechanisms by which it can regulate the excitability of SCN neurons including pre-synaptic regulation of glutamate release, as well as the post-synaptic regulation of GluRs, activation of voltage-sensitive Ca^2+ ^channels [17] and release of Ca^2+ ^from intracellular stores [16,34].

In the present study, we examined the concentration dependence of PACAP evoked changes in Ca^2+ ^transients and PACAP enhancement of AMPA currents. We did not find significant measurable changes at the 0.1 and 1.0 nM PACAP concentrations. We focused on the nanomolar concentration range as we wanted to use the lowest possible concentrations that produced physiological effects. Previous studies have found physiological effects of PACAP on SCN neurons in the nanomolar concentration range [23,44]. We did not explore the micomolar concentrations though PACAP at these concentrations has been shown to alter glutamate induced regulation of *mPer *and phase shifts of neural activity rhythms [19,24].

There is also evidence that PACAP actions on the circadian system can vary with the phase of the daily cycle. When measur ing neural activity rhythms from rat brain slices, administration of PACAP in micromolar concentrations enhanced the phase shifting effects of glutamate in the early night but had the opposite effect in the late night [24]. Furthermore, the administration of anti-PACAP antibodies into the cerebral ventricles of hamsters was reported to enhance light-induced phase advances [24] and attenuate light-induced phase delays [27]. Light stimulation in the early night resulted in larger phase delays in PAC1 -/- mice accompanied by a reduction in light-induced *mPer *and *c-Fos *expression [21]. In the late night, photic stimulation of the PAC1 -/- mice produced phase delays instead of the usual phase advances and was not associated with any changes in *mPer *or *Fos*. These studies have led to the proposal that PACAP's effects on the light-input to the circadian system are phase-dependent in that PACAP will enhance light-induced phase delays but inhibit light-induced phase advances [24,51]. In contrast, a recent study found that PACAP-deficient mice exhibited significantly attenuated phase advances but light-induced phase delays were only slightly diminished [30]. In our own work, we found PACAP -deficient mice exhibited significant impairment in the magnitude of the response to brief light exposures with both light-induced phase delays and advances of the circadian system impacted [31]. The present physiological data indicate that PACAP enhances AMPA and NMDA currents in the early night. Thus our data lead us to propose that PACAP plays a role modulating the response of SCN neurons to glutamatergic stimulation and is required for normal light-induced synchronization of the circadian system.

## Conclusion

In our view, PACAP acts through multiple mechanisms to enhance glutamatergic input to the SCN and that loss of this modulation results in a loss of sensitivity to photic stimulation during the night. First, our finding that PACAP increases sEPSC frequency and the anatomical finding that PAC1 receptors are expressed in retinal ganglion cells [35] raises the possibility that PACAP may act presynaptically to regulate the release of glutamate onto SCN neurons. In addition, PACAP enhances both NMDA-evoked and AMPA-evoked currents in SCN neurons. Our evidence suggests that PACAP regulation of the magnitude of AMPA currents is dependent upon PAC1 receptors and the adenylyl cyclase-signalling cascade. Interestingly, a recent study found evidence suggesting that the enhancement of AMPA mediated synaptic transmission in the SCN is sufficient to increase the magnitude of light-induced phase delays [52]. Finally, PACAP can increase Ca^2+ ^by itself as well as enhance AMPA- and glutamate -evoked increases in Ca^2+ ^[16,17,34]. L ikely through these mechanisms, PACAP can regulate *mPer *expression in SCN neurons [18,19]. Certainly, a better understanding of the cellular mechanisms by which PACAP regulates SCN neurons is a critical step toward the development of a mechanistic explanation for the role of this peptide in the circadian system.

## Methods

### Brain slice preparation

The University of California, Los Angeles Animal Research Committee approved the experimental protocols used in this study (protocol #: 1997-027-23A). Briefly, mice and rats were killed by decapitation, brains dissected and placed in cold oxygenated ACSF containing (in mM) NaCl 130, NaHCO_3 _26, KCl 3, MgCl_2 _5, NaH_2_PO_4 _1.25, CaCl_2 _1.0, glucose 10 (pH 7.2–7.4). After cutting slices (Microslicer, DSK Model 1500E) from areas of interest, transverse sections (350 μm) were placed in ACSF (25–27°C) for at least 1 h (in this solution CaCl_2 _is increased to 2 mM, MgCl_2 _is decreased to 2 mM). Slices are constantly oxygenated with 95% O_2_-5% CO_2 _(pH 7.2–7.4, osmolality 290–310 mOsm).

### Infrared Differential Interference Contrast (IR DIC) Videomicroscopy

Slices were viewed with an upright compound microscope (Olympus BX50), using a water immersion lens (40X) and DIC optics. They were illuminated with near IR light by placing an IR bandpass filter (750–1050 nm) in the light path. The image was detected with an IR-sensitive video camera (Hamamatsu C2400, Bridgewater, NJ) and displayed on a video monitor. A camera controller allowed analog contrast enhancement and gain control. Cells were typically visualized from 30–100 μm below the surface of the slice. In the present study, IR videomicroscopy was utilized to visualize cells within the brain slice and to limit some of the uncertainty as to the cell type. This imaging technique allowed us to clearly see the SCN and to exclude cells from the surrounding hypothalamic regions. In addition, we distinguished between dorsal and ventral SCN regions as previously described by Itri and Colwell [53].

### Whole cell patch clamp electrophysiology

Methods are similar to those described previously [32,33,53]. Briefly, electrodes were pulled on a multistage puller (Sutter P-97, Novato, CA). Electrode resistance in the bath was typically 3–6 MΩ. The standard solution in the patch pipette for measurement of spontaneous postsynaptic currents contains (in mM): K-gluconate, 112.5; Hepes, 10; MgATP, 5; NaCl, 4; EGTA, 1; MgCl_2_, 1; CaCl_2_, 0.5; GTP-Tris, 1; Leupeptin 0.1; Phosphocreatine 10. The pH was adjusted to 7.25–7.3 using KOH and the osmolality to 290–295 mOsm using sucrose. For measurement of amino-methyl proprionic acid (AMPA) and N-methyl-D-aspartate (NMDA) currents, Cs-methanesulfonate was used instead of K-gluconate in the pipette. Blockers of ionic channels were added to the bath solution to inhibit potassium (K^+^) currents (tetraethylammonium-chloride, TEA 10 mM replacing equal amounts of NaCl), Ca^2+ ^currents (cadmium, Cd^2+^, 25 μM), and sodium (Na^+^) currents (tetrodotoxin, TTX, 0.5 μM). Whole cell recordings were obtained with an Axon Instruments 200B amplifier and monitored on-line with pCLAMP (Axon Instruments, Foster City, CA). To minimize changes in offset potentials with changing ionic conditions, the ground path used a KCl agar bridge. The liquid junction potential was measured to be -14 mV. Cells were approached with slight positive pressure (2–3 cm H_2_O) and offset potentials were corrected. The pipette was lowered to the vicinity of the membrane keeping a positive pressure. After forming a high-resistance seal (2–10 GΩ) by applying negative pressure, a second pulse of negative pressure was used to break the membrane. Whole-cell capacitance and access resistance were neutralized and data acquisition was then initiated. Series and input resistance were monitored repeatedly by checking the response to small test pulses (10 mV) from the holding potential (-70 mV). Series resistance was not compensated and the maximal voltage error due to this resistance was calculated to be 6 mV. The access resistance of these cells ranged from 15–40 MΩ while the cell capacitance was typically between 6–18 pF.

Under voltage -clamp (Membrane potential = -70 mV) the holding current was monitored throughout the experiment. Spontaneous postsynaptic currents were analyzed using the MiniAnalysis program (Synaptosoft, Decatur, GA). The software was used to automatically record the number and peak amplitude of sEPSCs recorded in gap-free mode of pCLAMP software. Each automatically detected event was then manually checked to ensure that the baseline and peak were accurately determined. The mean frequency and amplitude of the EPSCs were then calculated for each neuron during 60–360 sec sampling periods.

### Calcium imaging

Methods are similar to those described previously [5,32]. In brief, a cooled charge-coupled device camera (Princeton Instruments, Monmouth Junction, NJ, Microview model 1317×1035 pixel format) was added to the Olympus fixed stage microscope to measure fluorescence. In order to load the calcium-indicator dye into cells, slices were incubated with membrane permeable fura2 AM (50 μM) at 37°C for 10 min. The fluorescence of fura2 was excited alternatively at wavelengths of 357 nm and 380 nm by means of a high-speed wavelength-switching device (Sutter, Lambda DG-4). Image analysis software (MetaFlour, Universal Imaging, Downingtown, PA) allowed the selection of several regions-of-interest within the field from which measurements were taken. In order to minimize bleaching, the intensity of excitation light and sampling frequency was kept as low as possible.

### Lighting condition

For the electrophysiology and calcium imaging studies, mice (C57BL/6) and rats (Sprague -Dawley) were maintained on a daily light-dark (LD) cycle consisting of 12 h of light followed by 12 h of dark at constant room temperature for at least 10 days prior to the experiment. It is already well established that cells in the SCN continue to show circadian oscillations when isolated from the animal in a brain slice preparation. The rodents were killed at the time that the lights would have turned off in the LD cycle, which is defined as Zeitgeber time (ZT) 12. The data from all animals were collected between ZT 15–17.

### Drug application

Solution exchanges within the slice were achieved by a rapid gravity feed delivery system utilizing an electronically-controlled valve. In our system, the effects of bath-applied drugs begin within 15 sec and are typically complete by 2 min. In this study, AMPA and NMDA were applied for 120 sec with maximal responses typically observed approximately 90 sec after start of treatment. PACAP, FSK, and H89 were applied for 240 sec and in some cases, these treatment were immediately followed by application of AMPA or NMDA.

The PACAP (1–38) was purchased from American Peptide Company (Sunnyvale, CA) whereas maxadilan and M65 were generously provided by Dr. E. Lerner (Harvard University, Cambridge, MA). The peptides were dissolved in distilled water as concentrated stock solutions. H89 was purchased from Calbiochem (La Jolla, CA) while all other chemicals were purchased from Sigma (St. Louis, MO). All glassware and perfusion lines were silanized using Sigma -Cote and blocked with 2% protenate solution (Baxter Healthcare Corp., Glendale, CA) to prevent unspecific binding of the peptides.

### Statistical analyses

Between group differences were evaluated using t-tests or Mann-Whitney rank sum tests when appropriate. Values were considered significantly different if *P *< 0.05. All tests were performed using SigmaStat (SPSS, Chicago, IL). In the text, values are shown as mean ± SEM. For the electrophysiology, each data point represents one neuron in a different slice. We sometimes recorded from two slices of the same animal. For the imaging experiments, Ca^2+ ^levels from 6–20 neurons were measured simultaneously. Each group of data is collected from at least 4 animals, with *n *representing the number of cells recorded.

## Abbreviations

adenylyl cyclase, AC

amino-methyl proprionic acid, AMPA

artificial cerebral spinal fluid, ACSF

2-amino-5-phosphonovalerate, AP5

cadmium, Cd^2+^

calcium, Ca^2+^

cesium, Cs^+^

6-cyano-7-nitroquinoxaline-2,3-dione, CNQX

dark-dark, DD

differential Interference Contrast, DIC

γ-aminobutyric acid, GABA

hertz, Hz

infrared, IR

light-dark, LD

magnesium, Mg^2+^

N-methyl-D-aspartate, NMDA

pituitary adenylyl cyclase activating peptide, PACAP

potassium, K^+^

retinohypothalamic Tract, RHT

sodium, Na^+^

suprachiasmatic nucleus, SCN

tetraethyl-ammonium chloride, TEA

tetrodotoxin, TTX

zeitgeber time, ZT

## Authors' contributions

SM carried out electrophysiological studies and participated in the design of all experiments. JI carried out the measurements of the sEPSCs. JH carried out the measurements of the NMDA currents. KG carried out the measurements of the manipulations of the AC signalling pathway. CSC carried out the measurements of calcium transients, participated in the experimental design, and drafted the manuscript. All authors read and approved the final manuscript.
